# Type–II superlattices base visible/extended short–wavelength infrared photodetectors with a bandstructure–engineered photo–generated carrier extractor

**DOI:** 10.1038/s41598-019-41494-6

**Published:** 2019-03-21

**Authors:** Arash Dehzangi, Ryan McClintock, Abbas Haddadi, Donghai Wu, Romain Chevallier, Manijeh Razeghi

**Affiliations:** Center for Quantum Devices, Department of Electrical and Computer Engineering, Northwestern University, Evanston, Illinois 60208 USA

## Abstract

Visible/extended short–wavelength infrared photodetectors with a bandstructure–engineered photo–generated carrier extractor based on type–II InAs/AlSb/GaSb superlattices have been demonstrated. The photodetectors are designed to have a 100% cut-off wavelength of ~2.4 μm at 300K, with sensitivity down to visible wavelengths. The photodetectors exhibit room–temperature (300K) peak responsivity of 0.6 A/W at ~1.7 μm, corresponding to a quantum efficiency of 43% at zero bias under front–side illumination, without any anti–reflection coating where the visible cut−on wavelength of the devices is <0.5 µm. With a dark current density of 5.3 × 10^−4^ A/cm^2^ under −20 mV applied bias at 300K, the photodetectors exhibit a specific detectivity of 4.72 × 10^10^ cm·Hz^1/2^/W. At 150K, the photodetectors exhibit a dark current density of 1.8 × 10^−10^ A/cm^2^ and a quantum efficiency of 40%, resulting in a detectivity of 5.56 × 10^13^ cm·Hz^1/2^/W.

## Introduction

Visible/extended short–wavelength infrared (Vis/e–SWIR) imaging is a non-destructive optical analysis technique that can be used to obtain information that would otherwise be unavailable with conventional visible light imaging alone^1,2^; for instance, it can be used to see hidden layers in cultural heritage objects. This technique can be used to distinguish and recognize materials, to enhance the visibility of faint or obscured features, to detect signs of degradation, and to study the effects of environmental conditions on objects. This same capability for extended spectral imaging can also be used by NASA for planetary sciences to study the atmospheres and surfaces earth and other nearby planetary bodies. Images in the e-SWIR spectrum are used in many satellites for remote sensing applications. The spectrum is well known for its sensitivity to moisture, which offers vital measures in remote sensing applications, such as leaf water content, plant water stress, the remote sensing of vegetation liquid water and forest fire burn severity^3–5^.

Precise quantification of spectral and spatial features with extended spectral imaging can also be implemented in medical applications such as to analyze spectral and colorimetric properties of the skin reliably and non-invasively for medical applications^6^. Images taken by Vis/e–SWIR light can offer the interchange between the reflection of visible light, which may be invisible for human eyes which can be an opening for a new domain of special effects or surrealistic colors in variety of materials^2,7^.

Vis/e–SWIR spectrum imagers are also advantageous for military applications such as, long range visibility, haze/fire penetration, maritime and ground target contrast^8^. The e-SWIR passes through small particles (haze or smoke) which is a key point for enhanced long range visibility in surveillance applications^5,9^, where longer wavelength of e-SWIR spectrum also can create better visibility since it is less affected by the Rayleigh scattering^5^.

For imaging application, multispectral imagers have been presented in which by combinations of Vis/e–SWIR focal plan array (FPA) camera system with thermal infrared (TIR) cameras creates systems with advance interpretable images rather than just a thermal ones^10^.

So far, a variety of material systems have been used for visible/infrared imaging; with their own advantages and disadvantages. For example, silicon is the main semiconductor material that is used for visible light and near infrared (NIR) image sensors; but it has a fixed cut-off wavelength of ~1.1 µm and it is an indirect bandgap semiconductor which makes it non–ideal for light detection. In_x_Ga_1−x_As based photodetectors, which are almost lattice matched to InP substrates, exhibited high performance devices^11^, however, the performance diminishes at longer wavelengths due to excess of mismatch–induced defects^12^. Photodetectors based on the more mature mercury–cadmium–telluride (HgCdTe) material system are able to cover the visible/e–SWIR spectral range with remarkable performance; nonetheless, complex fabrication procedure and material growth can make these sensors prohibitively expensive^13^.

As a developing material system, Type–II superlattices (T2SLs) have plenty of advantages for infrared detection and imaging including unique band gap engineering capability, lower costs for growth and manufacturing, suppression of auger recombination with reliable material uniformity over large grown area^14–16^. T2SLs have recently demonstrated coverage of the e−SWIR spectral region^17–24^, however, there have been no reports of visible/infrared photodetectors based on the T2SLs material system.

The main challenge for making a visible/e–SWIR photodetector is to suppress the photo–generated carrier recombination near the surface and inside the highly–doped contact. Because most of the visible photons (E > 1 eV) have much higher energy than the absorption region bandgap, they are strongly absorbed near the surface and would recombine either via surface dangling bonds or inside the highly–doped top contact of a conventional e–SWIR photodetector with a ~500 meV bandgap. Therefore, one needs to address these two issues in order to make a Vis/e–SWIR photodetector.

In order to address the surface recombination, a heterostructure with a larger bandgap top window/contact layer should be employed to help move the absorption away from the surface. Replacing the free surface of the Vis/e–SWIR absorption region with a lattice–matched large–bandgap window layer also reduces the interface recombination velocity of the narrower–bandgap absorption region. The second problem of absorption in the highly−doped contact can be solved by avoiding the need to highly dope the contact by extracting the photo−generated carriers from the device with a bandstructure–engineered photo–generated carrier extractor (BECX) instead of a conventional *pn* junction.

In present work using T2SL InAs/AlSb/GaSb based material, we combined these two schemes together and used a BECX as the window layer and engineered the band−alignment of this hetero−face to efficiently extract photo−generated carriers. The new BECX design allows the T2SL–based Vis/e–SWIR photodetector to operate with lower dark current densities with an optical response covering the visible light spectrum. In this letter, we report the demonstration of visible/extended short–wavelength infrared photodetectors based on type–II InAs/AlSb/GaSb superlattices with a bandstructure–engineered photo–generated carrier extractor.

## Results

**Figure 1:**
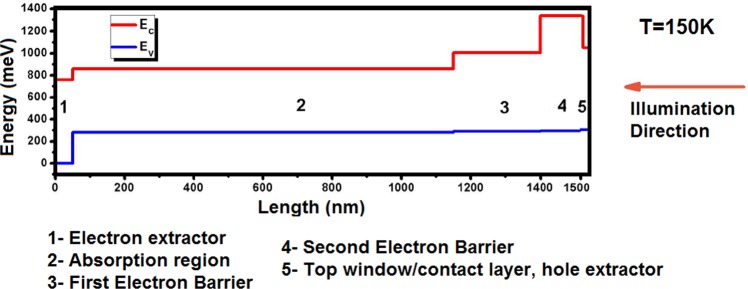
Schematic diagram of conduction (E_C_) and valence (E_V_) bands of the visible/e–SWIR photodetector at 150K, with a bandstructure–engineered photo–generated carrier extractor. Section 1, 2, 3, 4, and 5 of the device have ~760, 580, 715, 1040, and 800 meV bandgap, respectively.

Figure 1 demonstrates schematic diagram of conduction (E_C_) and valence (E_V_) bands of the visible/e–SWIR photodetector with a BECX, where section 1, 2, 3, 4, and 5 of the device have ~760, 580, 715, 1040, and 800 meV bandgap, respectively. The 1.1 μm–thick Vis/e–SWIR absorption region of the photodetector (section 2 in Fig. 1) is placed between two parts of the photo–generated carrier extractor. The extractor design extracts holes and electrons from the top and bottom of absorption region, respectively. The absorption region was chosen to have 6/1/5/1 mono–layers (MLs) of InAs/GaSb/AlSb/GaSb (unintentionally doped, *n*–type-10^14^ cm^−3^), respectively. The superlattice was designed using the empirical tight–binding model (ETBM)^25^. The photo−generated carrier transport inside the absorption region relies entirely on diffusion; thus, the new photo–generated carrier extractor does not require the applied bias which is required by other unipolar photodetector structures, such as nBn and pMp^17,26,27^; as such, it functions under zero bias like a conventional *pn* junction photodetector.

Each respective part of the photo–generated carrier extractor only allows extraction of either electrons or holes. The electron extractor (section 1 in Fig. 1) consists of 6/5 MLs of InAs/AlSb (*n*–type 10^18^ cm^−3^), respectively; which performs extraction of the electrons from the absorption region while it blocks the transport of holes and serves as the *n*−contact.

The combination of sections 3, 4, and 5 in Fig. 1 form the electron barrier and hole extractor. They create a digitally−graded energy profile in the valence band which generates a quasi–electric field and acts like a semi–*pn* junction. The first section of the electron barrier (Section 3) consists of 4/1/5/1 MLs of InAs/GaSb/AlSb/GaSb, (unintentionally doped, n–type-10^14^ cm^−3^), respectively. The middle section of the electron barrier (Section 4) was chosen to have 5/2 MLs of AlAs_0.10_Sb_0.90_/GaSb (p–type 10^17^ cm^−3^)^17^, respectively; and finally the last section (Section 5) consists of 10 MLs of GaSb (p–type 10^18^ cm^−3^). The thickness of section 4 should be high enough in order to block electron tunneling as much as possible, at the same time this section as a barrier, must be high enough to stop thermally excited electrons passing over the barrier (section 4) and so that there is negligible visible absorption inside this electron–barrier. The wide–bandgap of sections 3 and 4 together are able to reduce the generation/recombination (G-R) based dark current as well as some portion of the dark current which comes from the band–to–band tunneling and trap–assisted.

**Figure 2:**
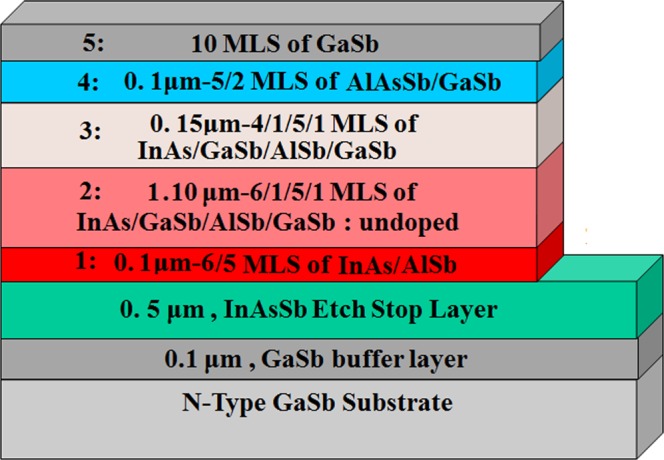
Schematic diagram of the e–SWIR photodetector with a bandstructure–engineered photo–generated carrier extractor and an etch stop layer on GaSb substrate.

After choosing the design of superlattice for separate parts of the photodetector the device was grown by solid source molecular beam epitaxy (SSMBE) with group III SUMO^®^ cells and group V valved crackers. The substrate used for the grown material was Te–doped GaSb wafer (*n*–type-10^17^ cm^−3^). The epitaxial growth begun by growing a 100 nm GaSb buffer layer followed by 500 nm *n*–doped InAs_0.91_Sb_0.09_ etch stop layer (10^18^ cm^−3^). The whole BECX structure with the thickness of ~1.5 µm was grown next as it is illustrated in Fig. 1. Figure 2 demonstrates the schematic diagram of the whole structure of the photodetector after the epitaxial growth.

**Figure 3:**
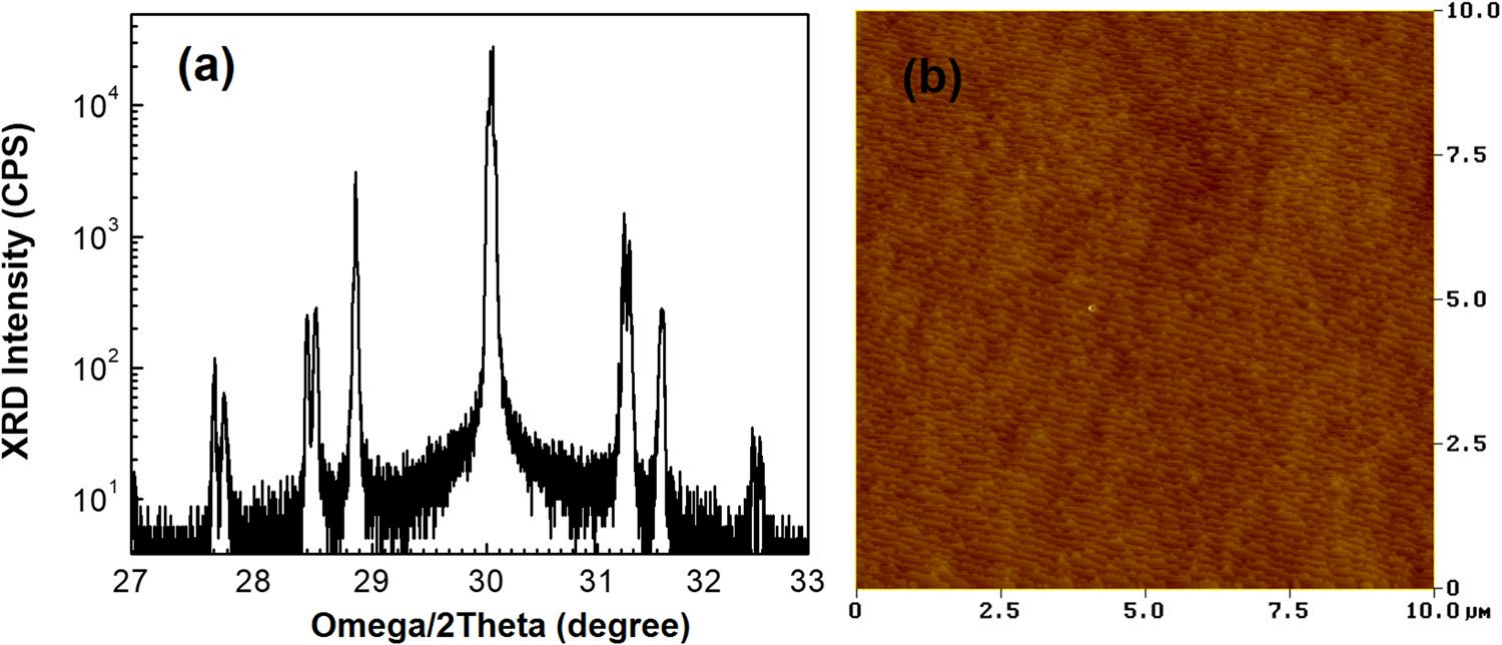
(**a**) Result for high–resolution X–ray diffraction of the grown material. (**b**) The AFM image for surface area of a 10 × 10 μm^2^ with the value of 0.86 Å for RMS roughness.

The material quality right after MBE growth was evaluated using high–resolution X–ray diffraction (HR–XRD) and atomic force microscopy (Fig. 3a,b). Analysis of the HR−XRD satellite peaks showed the overall periods of sections 1, 2, 3, and 4 were about 33, 45, 39, and 22 Å, respectively. The various superlattices that make up the structure are all lattice matched to the GaSb substrate to within 1000 ppm, which is in agreement with the superlattice designs. AFM results reveals high quality for surface morphology (clear atomic steps can be seen in Fig. 3b) with a small surface–roughness of 0.86 Å over a 10 × 10 μm^2^ area. This indicates that no structural degradation was introduced to the material due to presence of multilayer superlattices in BECX structure.

**Figure 4:**
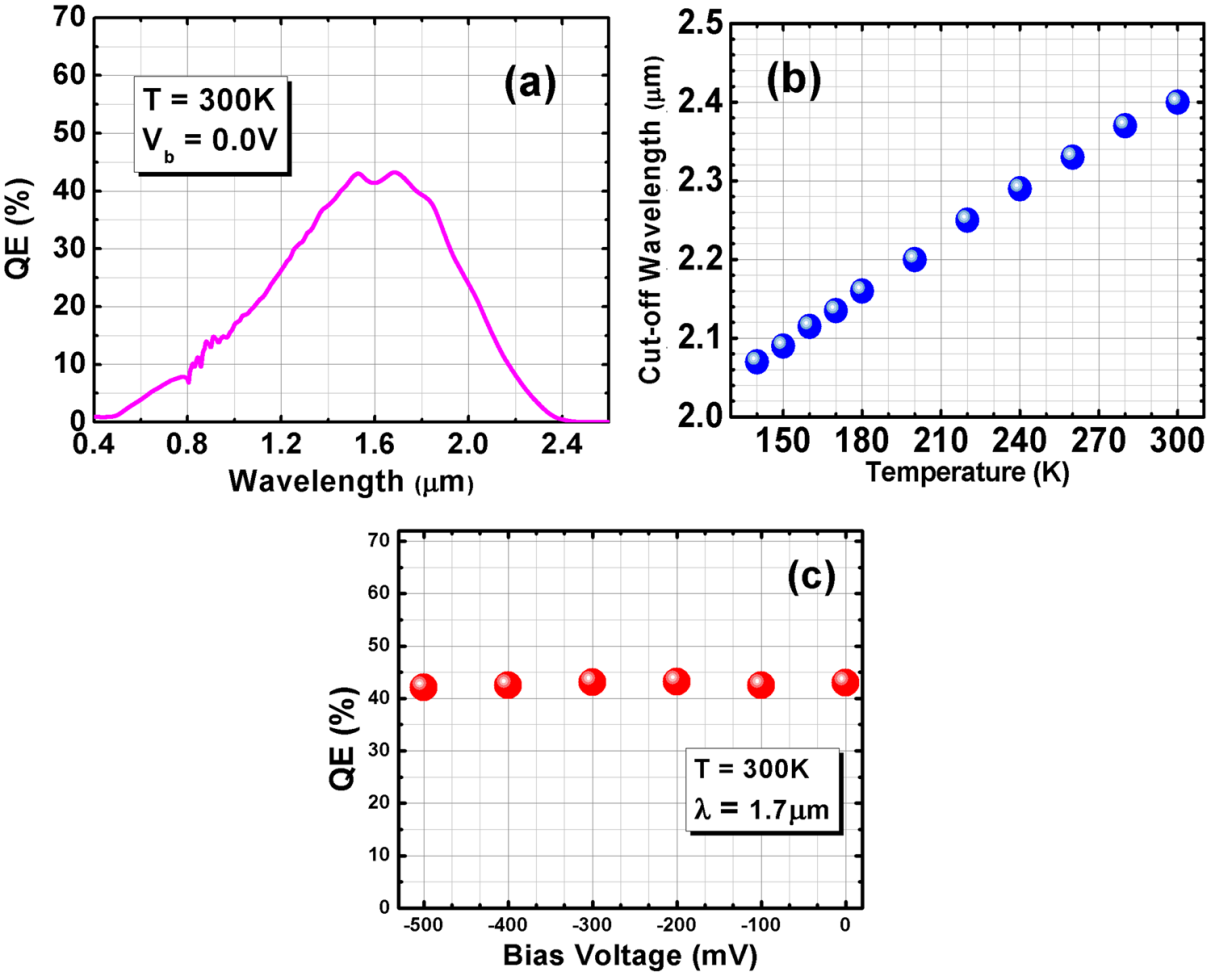
(**a**) Saturated quantum efficiency spectra of the photodetector at 300K in front-side illumination (no AR coating). (**b**) 100% Cut-off wavelength of the BECX photodetector vs. temperature. (**c**) Quantum efficiency vs. applied bias voltage at 1.7 μm at 300 K.

A calibrated 1000 °C blackbody source and Fourier transform infrared (FTIR) spectrometer (Bruker IFS 66 v/S) were implemented to carry out optical characterization and front–side–illuminated. During optical test no anti–reflection (AR) coating was applied on the BECX photodetectors. The optical performance of the photodetectors is shown in Fig. 4a. They exhibit a 100% cut-off wavelength of ~2.1 μm at 150 K (Fig. 4b) as predicted from the band structure calculations; the visible cut−on wavelength of the device is <0.5 µm. The %100 cut-off wavelength was defined as the wavelength that the optical response (QE spectrum) reaches to the measurement system’s noise floor. The device responsivity then peaks at 0.51 A/W, corresponding to quantum efficiency (QE) of 40% for this photodetector with a 1.10 μm–thick absorption region.

At 300K, the device responsivity peaks at 0.6 A/W at ~1.7 μm with a 100% cut–off wavelength at ~2.4 μm; and the QE of 43%. The abruptness of the band-edge absorption is proportional to the variation in superlattice period during epitaxial growth. As expected, the QE and responsivity spectra do not show any bias−dependency, Fig. 4c demonstrates QE values at different applied bias voltage at 1.7 μm in 300K. The decreased quantum efficiency at shorter wavelengths is caused by partial absorption of the visible/near infrared light in the hole extractor part of the device (sections 3, 4, and 5) where it does not contribute to the photo–current. This issue could be addressed in the future by adopting thinner top contacts or larger bandgap energies.

**Figure 5:**
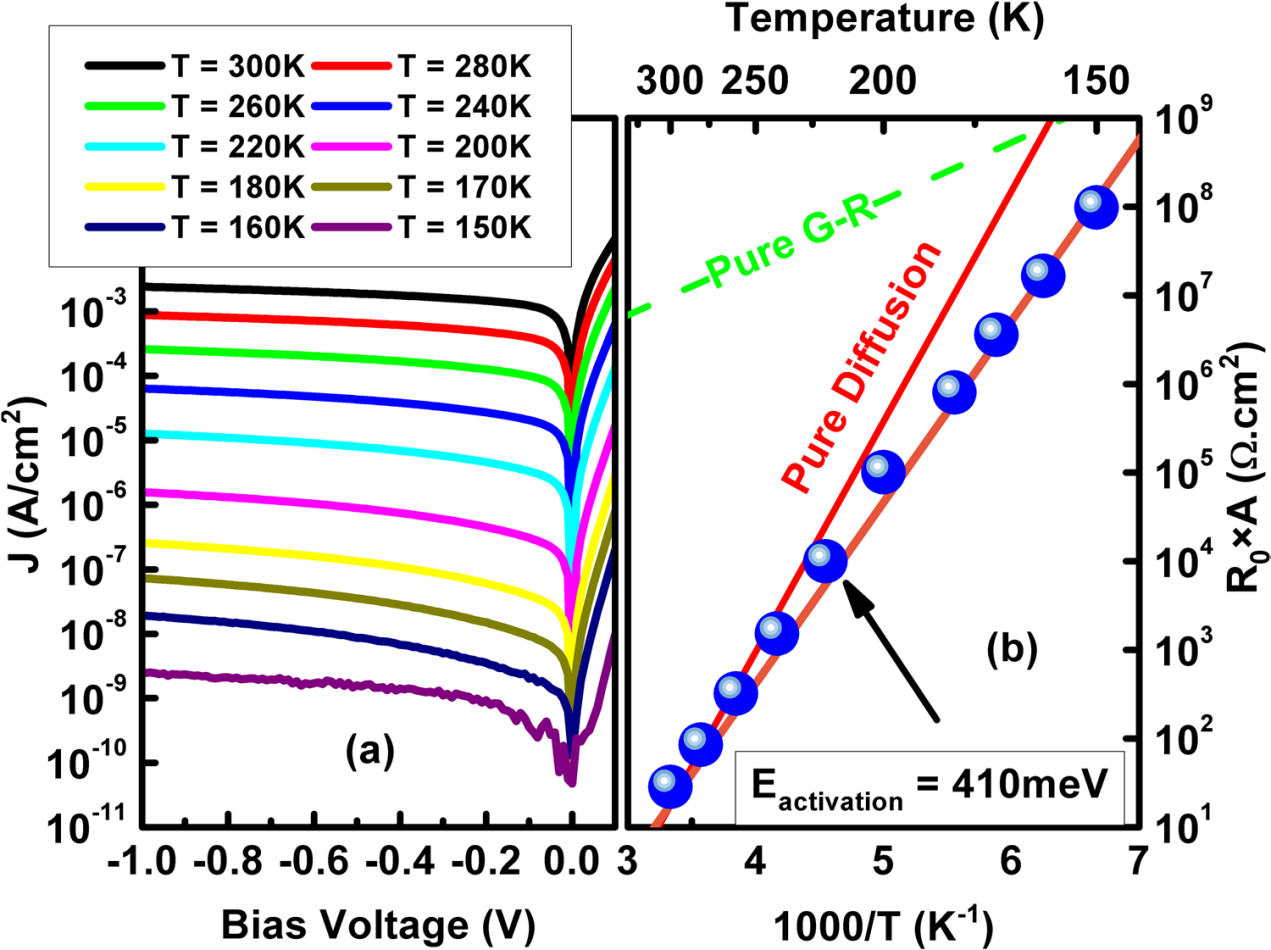
(**a**) Dark current density of BECX photodetector versus applied bias voltage, at different temperature. (**b**) Arrhenius plot of the differential resistance × area at zero bias (R_0_ × A). The dashed green and red lines present the expected diffusion and G-R limits, respectively.

Figure 5(a) shows the dark current density versus applied bias voltage of the BECX device at various temperatures spanning from 150 to 300K. At 150K, the device shows a dark current density of 1.8 × 10^−10^ A/cm^2^ under −20 mV applied bias, while at room temperature (T = 300K), the dark current density at −20 mV is 5.3 × 10^−4^ A/cm^2^. Figure 5(b) shows an Arrhenius plot^28^ of the differential resistance × area of the photodetector at zero bias (R_0_ × A) versus inverse temperature (1/T) from 150 to 300K. The dark current of the BECX photodetectors is nearly diffusion-limited at operating temperatures above 150K.

As it can be seen around 150K the deviation from pure diffusion suggests that the device is G-R limited at lower temperature. At low temperature, by changing the band gap, the G-R current starts to dominate the dark current due to the Shockley-Read-Hall (SRH) generation through trap states existed in the bandgap^29^. For instance, at low temperature, GaSb native defects inside the InAs/GaSb superlattice can produce SRH recombination centers which affect the carrier life time and increase the G-R current^30,31^.

Because of measurement system limitations, we could not measure device electrical performance below 150K to find the actual cross-over temperature (*T*_0_) of this device at which both the G-R and diffusion current become equal. However, the measurement result suggests that it should be below 150K; that is lower than *T*_0_ value for T2SL–based homo−junction photodiodes and nBn photodetectors operating in the e−SWIR region^17,18^, The BECX device architecture provide us to push down *T*_0_ to considerably lower operating temperatures with lower dark current compare to other T2SL-based e−SWIR photodetectors operating at the same temperatures. Consecutively, the BECX device can operate at a higher temperature with similar range of dark current. Beside the significant improvement electrical performance, the BECX design also has capability of being used in shallow-etched device geometry fabrication process, in which the mesa etch can terminate at the bottom of the hole extractor/window section and the smaller bandgap e−SWIR absorption region does not need to be etched, thus avoiding surface leakage. This could reduce the dark current further and may lead to more uniform focal plane arrays.

After performing optical and electrical testing, the specific detectivity (D^*^) of the BECX photodetector was calculated. The D* is defined as eq. (1):

1 $${D}^{\ast }={R}_{i}{[2q{J}_{c}+4{k}_{B}T/{R}_{A}]}^{-1/2}$$

where *R*_*i*_ is representing responsivity, q is the fundamental charge, *k*_*B*_ is the Boltzmann constant, *T* is temperature, *J*_*C*_ is dark current density, and *R*_*A*_ is differential resistance area product.

**Figure 6:**
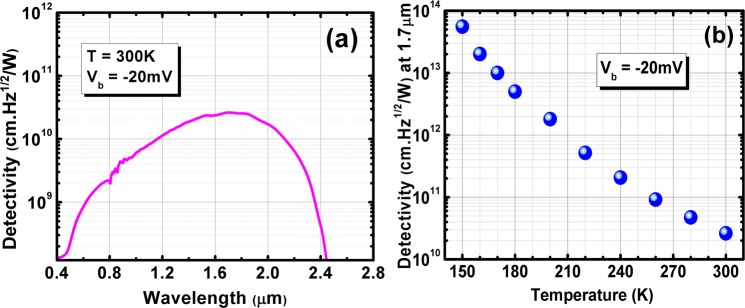
(**a**) Specific detectivity spectrum of BECX device at 300K (−20 mV applied bias). The device is front-side illuminated, without no AR coating. (**b**) The variation of the specific detectivity values of the device vs. temperature.

For 2π field-of-view (FOV) background condition, the device reveals a saturated dark current shot noise limited specific detectivity value of 5.56 × 10^13^ cm·Hz^1/2^/W at 150K and 4.72 × 10^10^ cm·Hz^1/2^/W (Fig. 6a) at 300 K with the applied bias of −20 mV, respectively. Figure 6b demonstrates the variation of the specific detectivity values of the device for different temperature. Table 1 shows the performance comparison of our photodetector (this work) with the other SWIR photodetector technologies.

**Table 1:** Performance comparison of this work with the other SWIR photodetector technologies.

	Cut-off Wavelength (μm)	Operating Temperature (°K)	Specific Detectivity (Jones)
This Work	2.4	300	4.72 × 10^10^
InGaAs^33^	1.7	300	5 × 10^12^
MCT^34^	2.4	250	5.92 × 10^12^
InAs/GaSb/AlSb T2SL^18^	2.2	300	1.7 × 10^10^

## Discussion and Conclusion

Based on InAs/AlSb/GaSb type–II superlattices, we have presented growth, design,, and characterization of high–performance visible/e−SWIR photodetectors, using a bandstructure–engineered photo–generated carrier extractor. The 100% cut–off wavelengths of theses BECX photodetectors were ~2.1 μm at 150K and ~2.4 μm at 300K. The visible cut−on wavelength of the BECX devices was measured to be <0.5 µm. The devices exhibited saturated quantum efficiency values of 40% and 43% at 150 and 300K, respectively. A dark current density of 1.8 × 10^−10^ A/cm^2^ was measured at 150K, under −20 mV applied bias. The optical and electrical characterization device has led us to achieve the specific detectivity of 5.56 × 10^13^ cm·Hz^1/2^/W at 150K. The dark current density value at 300K was 5.3 × 10^−4^ A/cm^2^ under −20 mV bias, which led to a specific detectivity of 4.72 × 10^10^ cm·Hz^1/2^/W.

The bandstructure–engineered photo–generated carrier extractor design has made it possible for T2SL–based e−SWIR to reach lower dark current densities and cover the visible light spectrum in its optical response. Furthermore, considering the benefits of unlimited charge integration capability for recent digital readout integrated circuit devices, the BECXs photodetector can be an excellent alternative to be used for high–performance room–temperature imaging systems. This progress can open the prospect of implementing T2SL–based visible/e−SWIR photodetectors in high–performance focal plan array infrared imaging systems and makes T2SL a feasible candidate for current state–of–the–art infrared detection and imaging technologies.

## Methods and Fabrication

For the fabrication of the device, the material after the growth was processed using standard photolithography into a set of mesa–isolated BECX photodetectors. The processed photodetectors have the areas spanning from 7.85 × 10^−5^ to 1.26 × 10^−3^ cm^2^. The detail about processing was already reported in somewhere else^32^. The photodetectors were left unpassivated but in order to minimize the surface leakage special attention was paid to ensure the surfaces were kept clean and minimal surface oxidization occurred. A 68–pin leadless ceramic chip carrier was wire–bonded to photodetectors for further electrical and optical characterization. For testing the devices at different temperature range (140 to 300K) a cryostat chamber was used.
